# Kinin B1- and B2-Receptor Subtypes Contract Isolated Bovine Ciliary Muscle: Their Role in Ocular Lens Function and Intraocular Pressure Reduction

**DOI:** 10.3390/ph17111501

**Published:** 2024-11-08

**Authors:** Najam A. Sharif, Madura Kulkarni-Chitnis, Anthonia Okolie, Ya Fatou Njie-Mbye, Sunny E. Ohia

**Affiliations:** 1 Department of Pharmaceutical Sciences, College of Pharmacy and Health Sciences, Texas Southern University, Houston, TX 75207, USAyafatou.njie-mbye@tsu.edu (Y.F.N.-M.); sunny.ohia@tsu.edu (S.E.O.); 2 Singapore Eye Research Institute (SERI), Singapore 169856, Singapore; 3 Institute of Ophthalmology, University College London (UCL), London WC1E6BT, UK; 4 Department of Ophthalmology, Imperial College of Science and Technology, St. Mary’s Campus, London SW72AZ, UK; 5 Department of Pharmacy Sciences, Creighton University, Omaha, NE 68178, USA; 6 Department of Pharmacology and Neuroscience, University of North Texas Health Sciences Center, Fort Worth, TX 76107, USA; 7 Department of Ophthalmology, Eye-ACP Duke-National University of Singapore Medical School, Singapore 169857, Singapore

**Keywords:** ciliary muscle, bradykinin, receptor subtypes, B1 receptors, B2 receptors, kinin, pharmacology, IOP regulation

## Abstract

**Background:** Bradykinin is an endogenously produced nonapeptide with many physiological and pathological functions that are mediated by two pharmacologically defined receptor subtypes, B1- and B2-receptors. Current studies sought to characterize the functional bradykinin (BK) receptors present in freshly isolated bovine ciliary muscle (BCM) using an organ-bath tissue contraction system. **Methods:** Cumulative longitudinal isometric tension responses of BCM strips (4–5 mm) were recorded before and after the addition of test compounds to BCM strips hooked up to an isometric strain gauge transducer system. **Results:** BK and its analogs (7–11 concentrations) contracted BCM in a biphasic concentration-dependent manner. The first high affinity/potency phase accounted for 40–60% of the maximal contraction by each of BK (potency, EC_50_ = 0.9 ± 0.3 nM), Lys-BK (EC_50_ = 0.7 ± 0.1 nM), Met-Lys-BK (EC_50_ = 1 ± 0.1 nM), Hyp3-BK (EC_50_ = 1 ± 0.2 nM), RMP-7 (EC_50_ = 3.5 ± 0.5 nM), and Des-Arg^9^-BK (EC_50_ = 10 ± 0.4nM) (mean ± SEM, n = 3–8). The second lower activity phase of contraction potency values for these peptides ranged between 100 nM and 3 µM. In the presence of a selective B1-receptor antagonist (R715; 0.1–10 µM), the concentration–response curves to Des-Arg9-BK (B1-receptor agonist) were still observed, indicating activation of B2-receptors by this kinin. Likewise, when B2-receptors were completely blocked by using a B2-selective antagonist (WIN-64338; 1–10 µM), BK still induced BCM contraction, now by stimulating B1-receptors. **Conclusions:** This agonist/antagonist profile of BCM receptors indicated the presence of both B1- and B2-receptor subtypes, both being responsible for contracting this smooth muscle. The BCM kinin receptors may be involved in changing the shape of the ocular lens to influence accommodation, and since the ciliary muscle is attached to the trabecular meshwork through which aqueous humor drains, endogenously released kinins may regulate intraocular pressure.

## 1. Introduction

The nine amino acid-containing peptide bradykinin (BK) and the octapeptide Des-Arg^9^-BK are naturally produced and released in many tissues of the mammalian body where they perform numerous functions [1,2,3]. Amongst the deleterious roles, BK and Des-Arg^9^-BK release inflammatory cytokines and induce edematous conditions and pain. However, this BK is also involved in beneficial activities such as tissue contraction, hormone secretion, cell proliferation, and cellular protection in a direct or indirect manner [1,2,3]. BK interacts with two major receptor subtypes, B1 and B2, which possess a higher affinity for and potency at the B2 receptor whose activity can be selectively blocked by the B2-selective antagonist WIN-64338 [1,2,3]. Conversely, Des-Arg^9^-BK is a selective agonist for the B1 receptor whereas R715 behaves as a B1 receptor selective antagonist [1,2,3]. While the mammalian eye is an immune-privileged organ, a robust kallikrein/kinase and kininergic system exists, which locally generates BK and other fragments of the peptide as determined by immunohistochemical and molecular biological techniques [2,4,5,6,7]. Furthermore, the latter endogenous peptides interact with B1- and B2-receptors, with the B2-type being constitutively expressed while B1-type receptors are present at a low level in most cells but are inducible in the presence of pro-inflammatory cytokines, endotoxins, or after tissue injury or trauma [1,2,3]. Many ocular tissues express B2-receptors in the membranes of kinin-responsive cells such as corneal and conjunctival epithelial cells, non-pigmented ciliary epithelial cells, ciliary muscle cells, and trabecular meshwork cells [2,4,5,6,7,8], although the mRNAs for the B1-receptors have also been reported in some of these cells [4,6]. However, both the B1- and B2-subtype receptors mediate the biological functions of BK and its analog peptides primarily via Gq-coupled signaling pathways, which utilize inositol phosphates, intracellular Ca^2+^ ([Ca^2+^]_i_), and diacylglycerol as second messengers within the cytoplasm, which can be quantitated as indices of receptor activation and utilized for biochemical/pharmacological characterization [2,5,6,7,8]. Additional receptor-mediated functional readouts include early response kinase (ERK1/2) and matrix metalloproteinase (MMP) gene and protein up-regulation and/or prostaglandin release in response to BK and its analogs [2,5,6,7,8].

Bradykinin has been reported to contract isolated smooth muscles from several mammalian species [9,10]. In the present study, our aim was to use a different functional readout to confirm the presence of and to pharmacologically characterize the kinin receptors in freshly isolated strips of bovine ciliary muscle (BCM) using a tissue contraction bioassay system. Such an organ-bath/tissue response system was previously utilized to characterize muscarinic cholinergic receptors [11,12,13], serotonin-2 receptors [14], endothelin receptors [15], and various other receptor types in isolated strips or rings of ocular tissues [16].

## 2. Results

**Figure 1:**
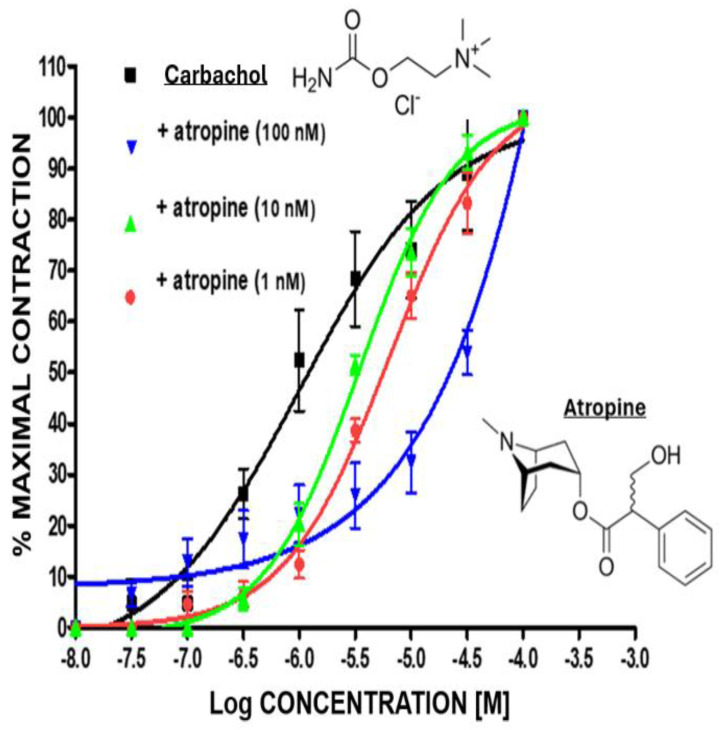
Carbachol contracts bovine ciliary muscle (BCM) and atropine inhibits the carbachol-induced contraction in vitro. The monophasic concentration-dependent profile of BCM contraction by carbachol was progressively shifted to the right in the presence of different concentrations of the muscarinic receptor antagonist, atropine. Mean ± SEM of n = 6–8 separate experiments using freshly isolated BCM strips.

All of the compounds tested yielded concentration-dependent contraction of BCM strips in vitro. The positive control agent, carbachol, always contracted the tissue, and its actions were concentration-dependently blocked by increasing concentrations of the muscarinic receptor antagonist, atropine, shifting the concentration-response curves of carbachol to the right (Figure 1). This profile confirmed the competitive antagonism properties of atropine, as has been documented in prior studies in various tissues, including BCM [11,12,13]. Furthermore, the concentration-response curve for carbachol, in the absence and presence of atropine, was monophasic, yielding a potency (EC_50_) value of 1 ± 0.07 µM (n = 6) for atropine (Figure 1) [11], with this being like values obtained in CM-derived cells and tissues [11,12,13]. The use of carbachol-induced BCM contraction was deemed necessary to ensure tissue viability in each experiment before any of the kinins were tested.

**Figure 2:**
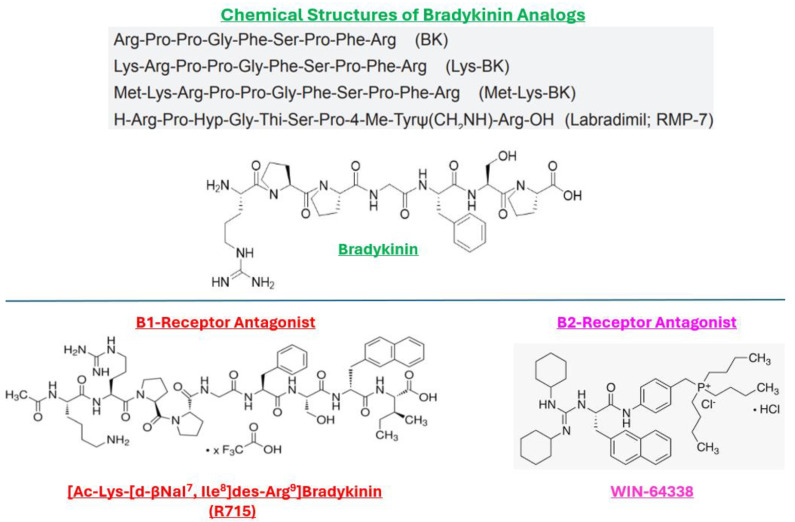
Chemical structures of bradykinin and its key analogs and those of the selective B1- and B2-receptor antagonists. The amino acid composition and linkage to form bradykinin and its analogs (linear peptides) are depicted in the upper panel. The lower panel shows the peptidic nature of R715 (B1-receptor antagonist; [17]) and the non-peptidic B2-receptor antagonist, WIN-64338 [1,2,3,8,18,19,20]. It must be noted that while WIN-64338 generally acts as a specific B2-receptor-selective antagonist, it has been shown to also block the cGMP-producing effects of Des-Arg^9^-BK, the B1-receptor agonist, in bovine aortic endothelial cells [21].

**Figure 3:**
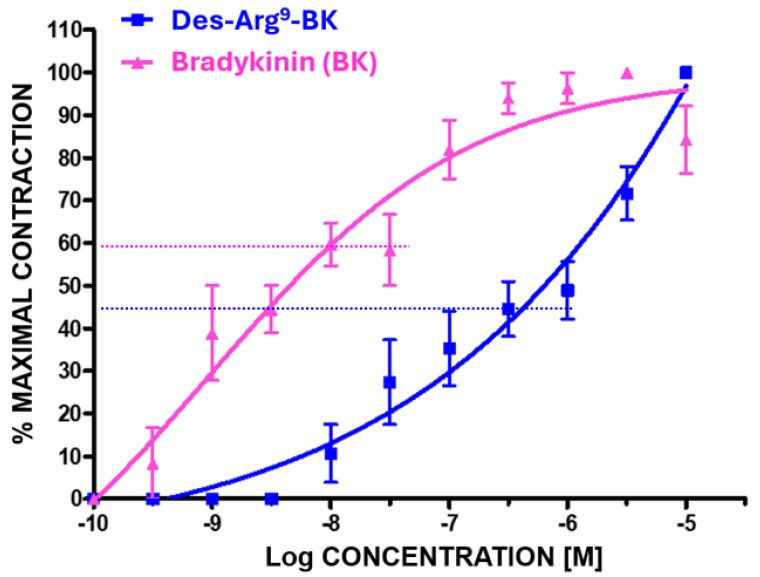
BCM contractions were induced by both the selective B1-receptor agonist (Des-Arg^9^-BK) and the B2-receptor agonist, bradykinin (BK), with different profiles of activity. Both peptides induced bi-phasic concentration-dependent contractile responses, yielding rather shallow graphs. These become apparent when the top of each high-potency phase of the graphs is delineated with a dotted line, as shown. Data derived from each phase of the concentration–response (C-R) curve yielded the relative functional potencies of the peptides at the two receptor sub-types. Thus, for example, BK exhibited a half-maximal response (EC_50_) at 0.9 nM concentration at the B2 receptor, while its potency at the B1 receptor was EC_50_ = 100 nM. Conversely, Des-Arg^9^-BK possessed an EC_50_ of 30 nM at the B1 receptor and an EC_50_ of 3 µM at the B2 receptor. Mean ± SEM of n = 3–8 independent experiments using strips of BCM isolated from fresh bovine eyes.

Regarding the actions of BK and its analogs (Figure 2), all produced concentration-dependent contractions of the BCM over the range of peptide concentrations used (0.1 nM to 10 µM) (Figure 3). All agonist kinin peptides displayed rather shallow concentration-response curves (e.g., Figure 3) as compared to the muscarinic receptor agonist, carbachol (Figure 1). The biphasic activity of the peptides was akin to that reported by Field et al. [17,18] in the guinea pig colon and trachea. The delineation of the high- (indicated by the dotted line representing the top of the first phase of the contraction, e.g., Figure 3) and low-potency components of the kinin-induced concentration-response curves yielded the potency values (EC_50_, half-maximal contractile concentration) for each of the two receptor sites. The data obtained from 3–8 experiments for each of the test compounds are shown in Table 1. The apparent rank order of potency of the peptides contracting the BCM strips at the high-affinity/potency site was Lys-BK ≥ BK ≥ Met-Lys-BK = Hyp^3^-BK > RMP-7 >> Des-Arg^9^-BK. Conversely, the compound rank order of potency at the low-affinity/potency site from the biphasic concentration-response curves (representing interaction with the B1-receptors) was BK = Met-Lys-BK = > Hyp^3^-BK > Lys-BK. However, due to the relative receptor subtype selectivity of Des-Arg^9^-BK, its interaction with the B1-receptor was represented by the high-potency site (EC_50_ = 30 nM), whereas the low-potency site representing the B2-receptor exhibited a low-potency component of the concentration-response curve (EC_50_ = 3000 nM) (Table 1).

**Figure 4:**
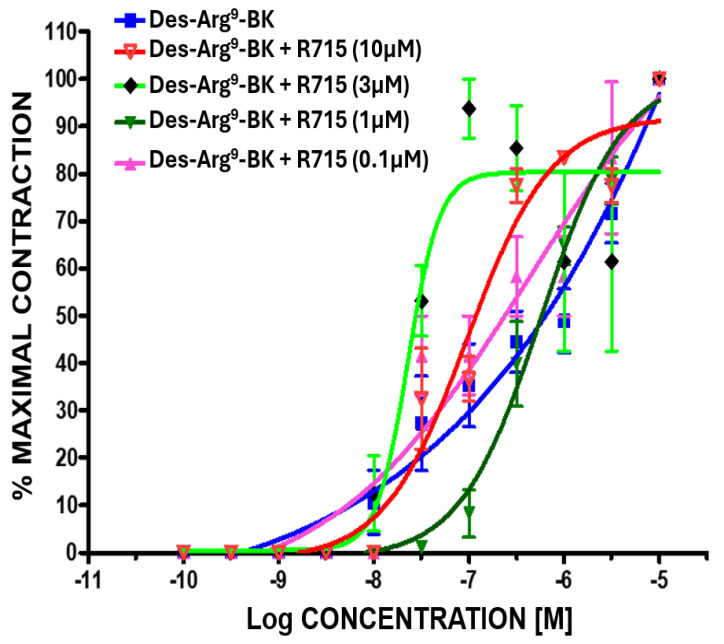
Complex effects of the selective B1-receptor antagonist (R715) on the concentration-response (C-R) curves of the B1-receptor agonist, Des-Arg^9^-BK, in the BCM contraction assay. Despite complete blockage of the B1-receptor by R715 (0.1–10 µM), Des-Arg^9^-BK still contracted the BCM strips but now by activating the B2-receptor. Mean ± SEM from up to 8 independent experiments using fresh strips of BCM isolated from bovine eyes.

**Figure 5:**
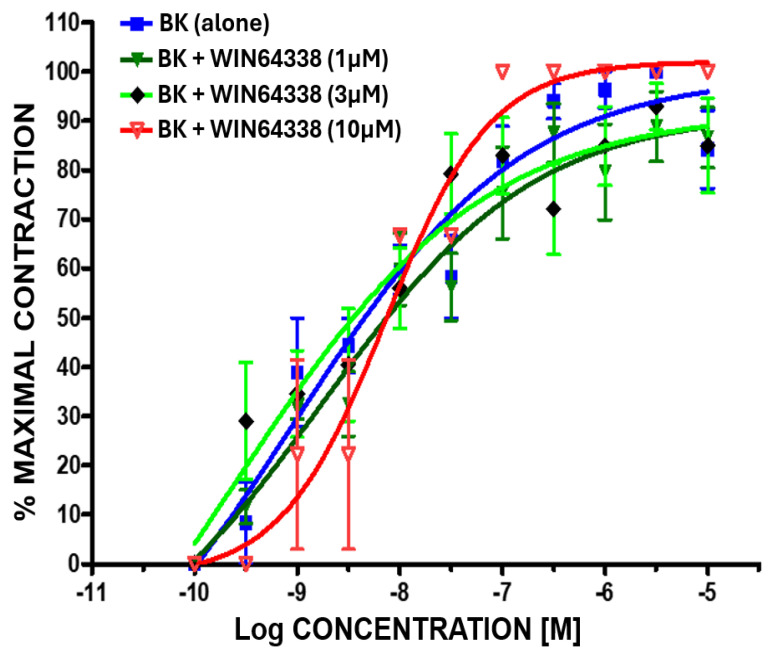
Complex effects of the selective B2-receptor antagonist (WIN-64338) on the concentration–response (C-R) curves of the B2-receptor agonist, BK, in the in vitro BCM contraction assay system. Even in the presence of the B2-receptor antagonist (1–10 µM), BK continued to contract the muscle, now by activating the B1-receptors. Mean ± SEM from up to 8 independent experiments using new strips of BCM isolated from freshly obtained bovine eyes.

By completely blocking the B1-receptor with a high affinity/potency B1-antagonist, R715 (0.1–10 µM [22]; Figure 2), Des-Arg^9^-BK was still allowed to stimulate the contraction of BCM strips (Figure 4). Likewise, completely blocking the B2 receptor with an antagonist for this subtype (WIN-64338 (1–10 µM [1,2,3,18]; Figure 2)) did not prevent BK from concentration-dependently contracting the BCM strips (Figure 5).

The functional contractile efficacies/potencies of BK and Des-Arg^9^-BK, two very receptor-subtype-differentiating kinins [1,2,3,19,20,23,24,25], in the BCM contraction bioassay, were compared with their activities in numerous tissues of different species (Table 2), either where the assays revealed biphasic actions of the peptides (e.g., guinea pig taenia caeci and trachea [17,18]) as in the BCM; or where only a single contractile receptor site was evident (e.g., B1-receptor in rat ileum [25]; or in rat aortic endothelium [22]; where Des-Arg^9^-BK exhibited the highest potency); or where only a B2-receptor-mediated contraction was observed (e.g., human umbilical vein, rat uterus, rat whole bladder) [1,2,3,18,20,23]; or where an undefined kinin receptor was described (e.g., in mouse/human bladder strips); or where BK had a low potency at the high-affinity binding site [10]. Additional functional assay data such as kinin-induced [Ca^2+^]_i_ mobilization, obtained from human ciliary muscle (hCM) cells and human trabecular meshwork (hTM) cells, which contain a single B2-receptor subtype, was also tabulated for comparison purposes (Table 2) [2,8].

## 3. Discussion

BK and Des-Arg^9^-BK are highly B2- and B1-receptor selective agonists, respectively, which have been used to pharmacologically characterize the subtypes of receptors activated by these peptides to mediate many functional activities in numerous diverse cells and tissues [1,2,3,7,8,19,20,23,24,25]. Additional subtype classification has been aided by specific B1- (e.g., R715) and B2-antagonists (e.g., WIN-64338) [1,2,3,7,8,20,23]. The agonist peptides have been reported to induce contractions of many tissues of different species and certain bioassays have been validated using agonist/antagonist profiles. For example, B1-receptors exist in the vascular smooth muscle of humans, rats, and rabbit aorta, renal artery, coronary artery [22,24], rabbit bladder [19], and rat ileum [25]. B2 receptors have been localized and characterized in a multitude of tissues and cells (e.g., in the brain: neurons within the thalamus, cerebral cortex, and hypothalamus; in the cardiovascular system: endothelial cells of the aorta, carotid arteries, and mesenteric arteries; other tissues: eye (e.g., ciliary muscle, trabecular meshwork, corneal and conjunctival epithelial cells), kidney, liver, lung, prostate, small intestine, uterus, etc.) [1,2,3,7,8,19,20,23,24,25]. Likewise, some tissues express both B1 and B2 receptors (e.g., guinea pig colon and trachea), as is evident from biphasic tissue contraction concentration–response curves for BK [17,18] (Table 2). The latter publications by Field et al. [17,18] clearly showed the low- and high-potency components of the contractile responses to bradykinin and thus directly support our observations of biphasic responses in the BCM preparation.

**Figure 6:**
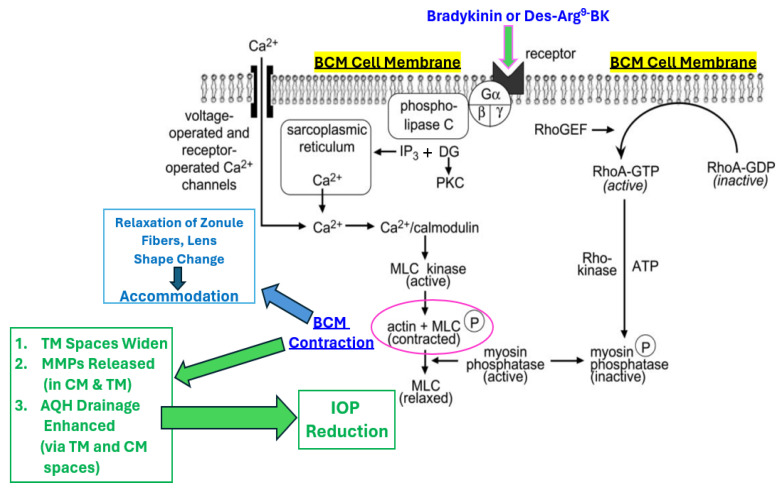
Schematic depiction of BCM smooth muscle cell contraction involves multiple intracellular mechanisms upon B1/B2 kinin receptor activation. BK receptor activation leads to the production of intracellular second messengers and consequential enhancement of various enzyme activities within the CM cells and connected tissues such as the TM and lens. These collective activities impact accommodative functions of the ocular lens and increase AQH dynamics to lower and control IOP.

Mechanistically, smooth muscle contraction is induced by kinin receptor-activated generation of inositol trisphosphate, amongst other inositol phosphates, that mobilizes and elevates [Ca^2+^]_i_, which binds to calmodulin and in turn activates myosin light chain (MLC) kinase. The latter process then leads to phosphorylation of the head of MLC, which eventually leads to cross-bridge cycling between actin and myosin ([16,26]; Figure 6). Myosin light chain phosphorylation is also regulated by MLC phosphatase, which removes the phosphate from MLC and promotes relaxation. Gα12/13-coupled receptor signaling involves activation of the small G-protein RhoA and consequently Rho-kinase, which inactivates MLC phosphatase leading to a sustained contraction ([10,20,26,27]; Figure 6). Although BK contracts smooth muscles in the body as described and referenced above, vasorelaxation of certain vascular beds by BK and Des-Arg^9^-BK has also been reported but this requires a relatively high basal tone of the tissues often due to the type(s) of innervation they receive and/or the prevailing local cellular environmental conditions [1,3].

In the current studies, both BK and Des-Arg^9^-BK contracted the BCM in a biphasic fashion, indicating the involvement of both B1 and B2 receptors in the smooth muscle contraction process. Other agonist peptides related to BK also elicited similar profiles of contractility, thereby substantiating the effects of BK and Des-Arg^9^-BK in this specific tissue. Furthermore, the role of both kinin receptor subtypes being responsible for contracting BCM in vitro was confirmed by sequentially recording the functional responses of the tissue in the presence of B1- and B2-receptor antagonists in numerous independent experiments. These collective observations are unique and novel and have been characterized for the first time in BCM. The agonist potency values for BK, Lys-BK, Hyp^3^-BK, Met-Lys-BK, and RMP-7 observed for the high-affinity BK-binding/potency site in the BCM contraction bioassay (0.7 to 3.5 nM EC_50_ values), corresponding to stimulation of B2-receptors (Table 1), compared well to those observed for other tissue contractile responses [1,3,17,18,19,22,23,24,25] (Table 2) and for the stimulation of second messenger production in various ocular cell-types involved in human CM and TM contraction (Table 2; [1,2,3,5,6,7,8]). Similarly, the ability of Des-Arg^9^-BK to induce BCM contraction via the high-affinity/potency binding site (1st phase of the contractile profile; Figure 3), and thus the B1-receptor (EC_50_ = 30 nM) (Table 1 and Table 2), compared closely with the rat ileum contraction potency of 18.6 nM (Table 2) [25]. Conversely, the second phase BCM contraction potency of Des-Arg^9^-BK (EC_50_ = 3000 nM), corresponding to its activation of the B2 receptor via the low-affinity/potency site, was closely similar to that obtained for human and rat uterus and for mobilizing [Ca^2+^]_i_ in human TM and human CM cells (Table 2) [2,5,6,8,26]. For contextual purposes, in recombinant B1 receptors expressed in host cells, BK exhibited a binding affinity (inverse binding inhibition constant; K_i_ values) of 200 nM (mouse), 5000 nM (rabbit), and 10,000 nM (human). Conversely, BK displayed K_i_ values at the cloned B2 receptor of 0.48 nM (mouse), 4.5 nM (rabbit), and 0.54 nM. Similarly, the K_i_ values of Des-Arg^9^-BK at cloned B1 receptors were 0.7 nM (mouse), 32 nM (rabbit), and 1930 (human) [1]. The K_i_ values of Des-Arg^9^-BK at the cloned B2-receptors were >1000 nM (rabbit), 6400 nM (mouse), and 8100 nM (human) [1]. Taken together, the agonist (and antagonist) profiles of kinin-mediated contraction of isolated BCM closely resemble the profiles of pharmacologically defined B1 and B2 receptors as reported for several tissues and cells expressing kinin receptors [1,2,3,7,8,17,18,19,20,22,23,24,25,26,27].

As to the functions of the B1- and B2-kinin receptor subtypes present in the BCM, several possibilities exist. The mammalian CM is composed of longitudinal and circular muscle fibers and is an important part of the eye that contributes to a person’s ability to view objects clearly at varying distances [16,28]. The contraction of the CM circular fibers primarily causes relaxation of the zonule fibers connected to the lens, rendering the latter more spherical [16,28]. Under these conditions, light refraction permits near vision. Obviously, the relaxation of the circular ciliary fibers allows far vision, and thus a change from contraction to relaxation of these fibers is responsible for accommodation performed by the lens located in the middle of the eye [16,28]. On the other hand, the muscle bundles of the radial part of the CM and the inner bundles of the longitudinal portion of the CM form tendons in their anterior insertion that are continuous with the extracellular matrix (ECM) of the TM beams and connect with the ECM of the scleral spur [16,28,29]. The latter contains elastic fibers and collagen, which are continuous with those of the core of the corneoscleral TM beams. Hence, due to this structural arrangement of these anterior uveal tissues, CM contraction pulls the scleral spur posteriorly and widens the spaces within the TM, thereby allowing the aqueous humor (AQH) to flow out of the anterior chamber and thereby lowering the IOP [16,29]. Therefore, endogenously released BK and its metabolism to Des-Arg^9^-BK within the CM can lead to contraction of the CM via B2 and B1 receptors, respectively, and thus regulation of intraocular pressure can be achieved by influencing the egress of AQH via the conventional outflow pathway [8,16,29]. The latter outflow pathway engaged by the CM accounts for 75–80% of the total drainage of AQH from the anterior chamber of the eye under normal conditions, which is progressively reduced in ocular hypertension and under glaucomatous circumstances [16,29]. Hence, locally released kinins can help regulate IOP and prevent glaucomatous optic neuropathy, a prevalent potentially blinding disease [2,4,5,6,8].

In addition to regulating conventional AQH outflow through CM contraction, the eye has developed another unconventional AQH drainage system that takes advantage of increasing the gaps between CM muscle bundles to affect IOP control [16,29]. In animals and human subjects that experience OHT/glaucoma due to blockage of the conventional outflow pathway by aberrantly depositing ECM in the TM and Schlemm’s canal area [16,29], AQH drainage via the CM spaces becomes more important. However, this requires the local generation and release of matrix metalloproteinases, which can digest the ECM in between the CM bundles to permit AQH efflux [16,29]. The BK receptors, in particular the B2 subtype present in the CM cells, can initiate the up-regulation of genes to enhance MMP production and thus help eliminate some of the ECM to increase uveoscleral outflow of AQH to lower IOP [2,8]. Indeed, such mechanisms have been shown to operate in response to the delivery of B2-receptor agonists to the anterior chamber (e.g., in rabbits after intravitreal injection of BK) [2,8] and in cynomolgus monkeys following topical ocular instillation of a non-peptide B2-receptor agonist whose IOP-lowering activity could be blocked by a B2-receptor antagonist [2,8]. Whether such IOP-lowering effects of BK and other kinins occur in cattle remains to be determined. Interestingly, however, Webb et al. [5] have demonstrated that BK was able to stimulate conventional outflow of AQH from anterior segments of bovine eyes in organ culture in a time-dependent manner that required the generation of MMPs. Such data strongly support the possibility that BK and its analogs would modulate IOP in vivo in cattle, and such studies are eagerly awaited.

In conclusion, the current studies have pharmacologically defined the presence of both B1- and B2-receptor subtypes in BCM using a variety of BK analogs and selective antagonists of each of the receptors [1,2,3,23]. These kinin receptor subtypes potently contract the smooth muscle within the BCM, which may be involved in accommodative and AQH dynamic/IOP regulatory functions. Since BK also contracts the pupillary sphincter muscle in rabbits [30] and lowers IOP in the same species [2,8] and given that non-peptide BK agonist lowers and controls IOP in monkeys via the B2-receptor [2,8], it is anticipated that the kinin receptors in BCM serve similar functions. However, further investigations are needed to demonstrate these effects of kinin receptor activation via the CM in cattle.

## 4. Materials and Methods

The methods utilized in the current studies were adapted from Lograno and Romano [13] and Ohia et al. [14], with some minor modifications. Fresh bovine eyeballs were obtained from a local abattoir (Fisher Ham and Meat Company, Houston, TX, USA) and transported to the laboratory in ice. Whole eyeballs were placed in warm oxygenated Krebs buffer solution for 30 min to ensure equilibration before dissection of the ciliary muscle. Briefly, the anterior segment was dissected out of whole eyeballs after an incision was made at the limbus. The anterior segment containing the cornea, anterior uvea, and lens was then inverted and the lens dislodged with a probe along with the attached vitreous humor. After removal of the vitreous humor and lens, the whole ciliary muscle (comprising both longitudinal and circular portions) was quickly isolated and dissected from the scleral spur and choroid. Muscle strips of 4–5 mm in length were cut and mounted in 25 mL organ baths containing oxygenated Krebs solution at 37 °C using a silk thread linked to an isometric strain gauge under a constant load of 1g tension. The tissues were then allowed to equilibrate for 30 min. Two ciliary muscle strips were prepared from each eyeball and four to eight strips were used for each series of experiments. The composition of the Krebs solution used (mM) was NaCl, 118; KCl, 4.8; CaCl_2_, 2.5; KH_2_PO_4_, 1.2; NaHCO_3_, 25; MgSO_4_, 2.0; dextrose, 10 and flurbiprofen, 0.003 (pH 7.4), all purchased from Sigma-Aldrich, St. Louis, MO (USA). Before any drug additions, the tissues were challenged with carbachol (10 µM) at least once to assess the functional state of the ciliary muscle strips and only those that yielded robust responses were subsequently utilized in the experiments. A robust response was characterized as a single contraction that consistently yielded a tension in the muscle induced by carbachol of 0.15–0.3 gm. Tissues that failed to elicit this level of response were excluded in the contractile studies of the bradykinin compounds. Tissue strips were then thoroughly rinsed with fresh buffer solution via a series of buffer additions and drainage activities to remove any residual muscarinic agonist, and the preload was readjusted prior to starting the actual study with kininergic ligands. Concentration–response studies employed 7–11 compound concentrations (0.1 nM to 10 µM) and data from multiple experiments were collected. Longitudinal isometric tension responses were recorded through an FTO3-transducer and displayed on a Polyview computer software 4.3 analyzer [14].

### 4.1. Chemicals

All of the compounds used were of the highest purity and were purchased from reputable vendors as described ahead. Bradykinin, Met-Lys-Bradykinin, Lys-Bradykinin, Hyp^3^-Bradykinin, RMP-7, Des-Arg^9^-Bradykinin, R715, and WIN-64338 were purchased from Tocris, Bio-Techne Corporation, Minneapolis, MN, USA. Atropine and carbachol were purchased from Sigma-Aldrich, Inc., St Louis, MO, USA. All other commonly utilized reagents/chemicals were also purchased from the latter company.

### 4.2. Data Analysis

The contractile responses obtained were expressed as gram tension developed and as percentages of the maximum response of each of the compounds. GraphPad Prism 4.0 Software (San Diego, CA, USA) was then used to construct the concentration–response curves for the kinins and to derive the half-maximal stimulation potencies (EC_50_) of each phase of the bi-phasic curves and the values tabulated as previously described [14].

## 5. Concluding Remarks

The data reported in this article represent novel findings pertaining to the possible functions of the bovine ciliary muscle when activated by bradykinin and its close structural peptidic analogs, including its metabolite Des-Arg^9^-BK. The availability and utility of the latter agonists and two well-known B1- and B2-receptor antagonists permitted the characterization of BK receptor subtypes involved in the contraction of the ciliary muscle. Unexpectedly, we discovered that BCM is the other unique tissue, along with the guinea pig colon and esophagus, that expresses functionally active B1- and B2-receptors, which promote the contractile process. These observations have extended our knowledge about BK receptor heterogeneity in an animal species that is not often utilized for ocular research purposes and thus opens up new avenues for future research using BCM preparations. The possible roles of endogenous or exogenously administered BK agonists for modulating intraocular lens function, perhaps combating myopia [31], and for treating elevated IOP, not just in monkeys [32] but in human subjects, warrant further studies in vitro and in vivo.

## Figures and Tables

**Table 1:** Functional potencies of BK and BK-based analogs at contracting bovine ciliary muscle strips in vitro.

Kinin Agonist	Reported Receptor Selectivity [1,2,3,18,20]	Max. Tension Generated During Contraction (gm Tension)	Functional Contractile Activity Potency (EC_50_)	
			High Affinity and Potency Phase	Low Affinity and Potency Phase
Bradykinin (BK)	B2 > B1	0.07 ± 0.01	0.9 ± 0.3 nM	100 ± 2.4 nM
Met-Lys-BK	B2 > B1	0.06 ± 0.01	1.0 ± 0.1 nM	100 ± 8.2 nM
Lys-BK	B2 ≥ B1	0.06 ± 0.01	0.7 ± 0.1 nM	500 ± 12.8 nM
Hyp^3^-BK	B2 > B1	0.04 ± 0.01	1.0 ± 0.2 nM	150 ± 9.0 nM
RMP-7	B2 > B1	0.07 ± 0.01	3.5 ± 0.5 nM	N/A
Des-Arg^9^-BK	B1 > B2	0.06 ± 0.01	30 ± 1.9 nM	3000 ± 72.0 nM

Data are mean ± SEM from 3–8 independent experiments using freshly obtained BCM strips and from tissue contraction concentration–response curves using 7–11 different concentrations (0.1 nM–10 µM) of each compound. N/A = not applicable.

**Table 2:** Ability of BK and Des-Arg^9^-BK to Contract Various Tissues of Different Species or to Mobilize Intracellular Ca^2+^ in human Ocular Cells.

Functional Potency Values in Various Tissues and Cells																		
Compound	BCM		GPTC		GPT		HUV		RUT		RWB		M/H BSTP		RI		hCM/hTM Cells	
	HP Site	LP Site	HP Site	LP Site	HP Site	LP Site	HP Site	LP Site	HP Site	LP Site	HP Site	LP Site	HP Site	LP Site	HP Site	LP Site	HP Site	LP Site
BK	0.9 nM	100 nM	3 nM	1000 nM	20 nM	4000 nM	9 nM	N/A	3 nM	N/A	90 nM	N/A	1200/ 5100 nM	N/A	4890 nM	N/A	2.4/1 nM	N/A
Des-Arg^9^-BK	30 nM	3000 nM	N/A	N/A	N/A	N/A	>1000 nM	N/A	>1000 nM	N/A	ND	ND	ND	ND	18.6 nM	N/A	4200/3600 nM	N/A

Data shown represent the mean functional potencies of the major kinins in contracting the smooth muscle contained in various tissues of multiple species, or their abilities to induce mobilization of intracellular calcium in human ciliary muscle (hCM) and human trabecular meshwork (hTM) cells in vitro, obtained from multiple experiments. BK is B2-receptor-selective, while Des-Arg^9^-BK is B1-receptor-selective. BCM = bovine ciliary muscle; GPTC = guinea pig tania caeca; GPT = guinea pig trachea; RUT = rat uterus strips; RWB = rat whole bladder; M/H BSTP = mouse/human bladder strips; RI = rat ileum; hCM = human ciliary muscle; hTM = human trabecular meshwork. HP site = high potency binding site; LP site = low potency binding site.

## Data Availability

The original contributions presented in the study are included in the article, further inquiries can be directed to the corresponding author.
